# 5-Meth­oxy-3-[(5-meth­oxy-1*H*-indol-3-yl)(phen­yl)meth­yl]-1*H*-indole

**DOI:** 10.1107/S1600536811045491

**Published:** 2011-11-05

**Authors:** P. Narayanan, K. Sethusankar, K. Ramachandiran, P. T. Perumal

**Affiliations:** a Department of Physics, RKM Vivekananda College (Autonomous), Chennai 600 004, India; b Organic Chemistry Division, Central Leather Research Institute, Adyar, Chennai 600 020, India

## Abstract

In the title compound, C_25_H_22_N_2_O_2_, the indole rings are individually almost planar [with maximum deviations of 0.0116 (19) and 0.0113 (18) Å] and are almost orthogonal to each other, making a dihedral angle of 84.49 (6)°. The benzene ring is inclined at 72.83 (9) and 80.85 (9)° with respect to the indole rings. In the crystal, mol­ecules are linked by N—H⋯O inter­actions into chains running parallel to the *c* axis. The crystal structure is further stabilized by C—H⋯π inter­actions.

## Related literature

For the biological activity and uses of indole derivatives, see: Bell *et al.* (1994 ▶); Ge *et al.* (1996 ▶). For related structures, see: Zhang *et al.* (2006 ▶, 2007 ▶). 
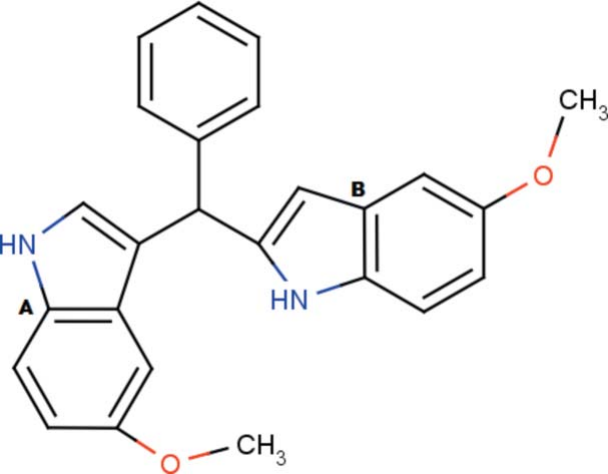


## Experimental

### 

#### Crystal data

C_25_H_22_N_2_O_2_
*M* *_r_* = 382.45
Monoclinic, 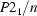

*a* = 9.1545 (4) Å
*b* = 10.5954 (6) Å
*c* = 21.1668 (13) Å
β = 93.679 (2)°
*V* = 2048.86 (19) Å^3^
*Z* = 4
Mo *K*α radiation
μ = 0.08 mm^−1^
*T* = 293 K
0.25 × 0.20 × 0.15 mm

#### Data collection

Bruker APEXII KappaCCD diffractometer
Absorption correction: multi-scan (*SADABS*; Bruker, 2008 ▶) *T*_min_ = 0.981, *T*_max_ = 0.988
16790 measured reflections
3087 independent reflections
2317 reflections with *I* > 2σ(*I*)
*R*_int_ = 0.034
θ_max_ = 23.7°

#### Refinement

*R*[*F* ^2^ > 2σ(*F* ^2^)] = 0.041
*wR*(*F* ^2^) = 0.119
*S* = 1.03
3087 reflections
264 parameters
H-atom parameters constrained
Δρ_max_ = 0.12 e Å^−3^
Δρ_min_ = −0.18 e Å^−3^

### 

Data collection: *APEX2* (Bruker, 2008 ▶); cell refinement: *SAINT* (Bruker, 2008 ▶); data reduction: *SAINT*; program(s) used to solve structure: *SHELXS97* (Sheldrick, 2008 ▶); program(s) used to refine structure: *SHELXL97* (Sheldrick, 2008 ▶); molecular graphics: *ORTEP-3* (Farrugia, 1997 ▶); software used to prepare material for publication: *SHELXL97* and *PLATON* (Spek, 2009 ▶).

## Supplementary Material

Crystal structure: contains datablock(s) global, I. DOI: 10.1107/S1600536811045491/pv2461sup1.cif

Structure factors: contains datablock(s) I. DOI: 10.1107/S1600536811045491/pv2461Isup2.hkl

Supplementary material file. DOI: 10.1107/S1600536811045491/pv2461Isup3.cml

Additional supplementary materials:  crystallographic information; 3D view; checkCIF report

## Figures and Tables

**Table 1:** Hydrogen-bond geometry (Å, °) *Cg*2, *Cg*3, *Cg*4 and *Cg*5 are the centroids of the N2/C17–C20, C1–C6, C10–C15 and C19–C24 rings, respectively

*D*—H⋯*A*	*D*—H	H⋯*A*	*D*⋯*A*	*D*—H⋯*A*
N1—H1*A*⋯O2^i^	0.86	2.09	2.904 (2)	158
C25—H25*B*⋯*Cg*2^ii^	0.96	2.95	3.515 (3)	119
C16—H16*C*⋯*Cg*3^iii^	0.96	2.90	3.508 (3)	122
N2—H2*A*⋯*Cg*4^iv^	0.86	2.49	3.3149 (19)	160
C3—H3⋯*Cg*5^v^	0.93	2.60	3.470 (2)	157

Symmetry codes: (i) 
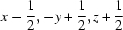
; (ii) 
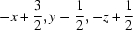
; (iii) 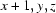; (iv) 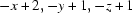; (v) 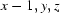.

**Fig. 1.**
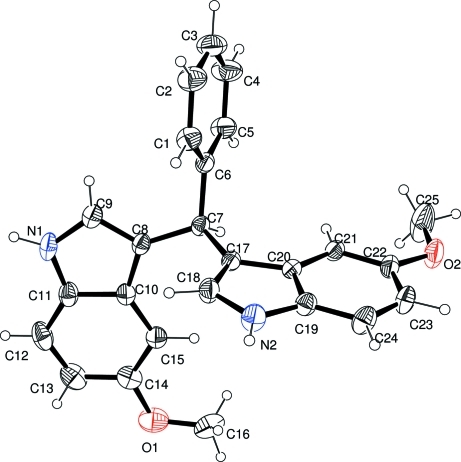
The molecular structure of the title compound with the atom numbering scheme; displacement ellipsoids are drawn at 30% probability level.

**Fig. 2.**
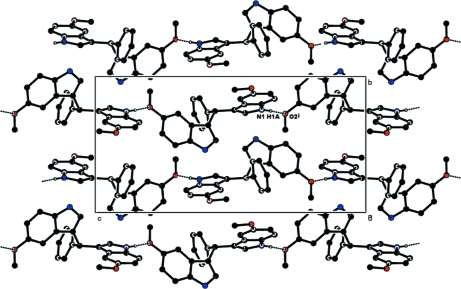
The packing arrangement of the title compound viewed down the a axis. The dashed lines indicate N—H···O intermolecular interactions resulting in chaims of molecules running parallel to the c axis.

## supplementary crystallographic information

### Comment

The synthesis of bis-indolylalkanes (BIAs) has been of considerable interest because of their occurance in various natural products posessing biological activities and usefulness in drug design (Bell, *et al.* 1994). These compounds also inhibit the proliferation of both estrogen dependent and independent cultured breast tumor cells (Ge, *et al.* 1996). In this paper, we present the synthesis and crystal structure of the title bis-indolylalkane derivative.

The title compound (Fig. 1) comprises a benzene ring and two methoxy indole rings connected through a carbon atom C7. The bicyclic indole rings, are individually planar with maximum deviations of 0.0116 (19)Å for C10 atom, in ring A (N1/C8–C15) and -0.0113 (18)Å for N2 atom, in ring B (N2/C17–C24). The indole rings are almost orthogonal to each other, with a dihedral angle of 84.49 (6)°. The deviations of methoxy group carbon atoms C16 and C25 from the rings A & B, are -0.118 (3)Å and -0.100 (3) Å, respectively.

The benzene ring (C1–C6) is inclined at 72.83 (9)° and 80.85 (9)°, with the indole rings A & B, respectively. The angles around atom C7 [C8–C7–C17 = 113.07 (14)°, C8–C7–C6 = 112.52 (14)° and C17–C7–C6 = 111.53 (15)°] deviate significantly from the ideal tetrahedral values which may be a result of steric interactions between benzene ring and the indole rings.

In the crystal packing, the molecules are linked by N—H···O intermolecular interactions into infinite chains running parallel to the *c* axis (Fig. 2). The crystal structure is further stabilized by C—H···*Cg* interactions where *Cg*2, *Cg*3, *Cg*4 and *Cg*5 are the centers of gravity of rings (N2/C17–C20), (C1–C6), (C10–C15) and (C19–C24), respectively (Table 1).

### Experimental

To benzaldehyde (1 mmol) in CH_2_Cl_2_ (10 ml) was added KHSO_4_ (30 mol%) and the mixture was stirred for 5 min. Methoxyindole (2 mmol) was added to the mixture and the stirring was continued following the progress of the reaction by TLC. After completion of the reaction, the reaction mixture was extracted with ethyl acetate (3 *x* 10 ml), dried over anhydrous sodium sulfate, filtered, concentrated under reduced pressure and the residue was column chromatographed over silica gel using EtOAc/Petroleum ether (1:19) as eluent to get the pure product, the title compound, which was re-crystallized by slow evaporation of its solution in ethyl acetate, resulting in single crystals, suitable for XRD studies.

### Refinement

The hydrogen atoms were placed in calculated positions with C–H = 0.93 to 0.98 and N–H = 0.86 Å and refined in the riding model with fixed isotropic displacement parameters: *U*_iso_(H) = 1.5*U*_eq_(C) for methyl groups and *U*_iso_(H) = 1.2*U*_eq_(C,*N*) for others. The crystal did not diffract beyond θ =23.7 ° as its mosaicity was quite high and a low temperature facility was not used for intensity data collection.

### Crystal data

**Table:**

C_25_H_22_N_2_O_2_	*F*(000) = 808
*M**_r_* = 382.45	*D*_x_ = 1.240 Mg m^−^^3^
Monoclinic, *P*2_1_/*n*	Mo *K*α radiation, λ = 0.71073 Å
Hall symbol: -P 2yn	Cell parameters from 3087 reflections
*a* = 9.1545 (4) Å	θ = 2.2–23.7°
*b* = 10.5954 (6) Å	µ = 0.08 mm^−^^1^
*c* = 21.1668 (13) Å	*T* = 293 K
β = 93.679 (2)°	Block, brown
*V* = 2048.86 (19) Å^3^	0.25 × 0.20 × 0.15 mm
*Z* = 4	

### Data collection

**Table:**

Bruker APEXII KappaCCD diffractometer	3087 independent reflections
Radiation source: fine-focus sealed tube	2317 reflections with *I* > 2σ(*I*)
graphite	*R*_int_ = 0.034
ω and φ scans	θ_max_ = 23.7°, θ_min_ = 2.2°
Absorption correction: multi-scan (*SADABS*; Bruker, 2008)	*h* = −7→10
*T*_min_ = 0.981, *T*_max_ = 0.988	*k* = −11→11
16790 measured reflections	*l* = −22→23

### Refinement

**Table:**

Refinement on *F*^2^	Primary atom site location: structure-invariant direct methods
Least-squares matrix: full	Secondary atom site location: difference Fourier map
*R*[*F*^2^ > 2σ(*F*^2^)] = 0.041	Hydrogen site location: inferred from neighbouring sites
*wR*(*F*^2^) = 0.119	H-atom parameters constrained
*S* = 1.03	*w* = 1/[σ^2^(*F*_o_^2^) + (0.0627*P*)^2^ + 0.4003*P*] where *P* = (*F*_o_^2^ + 2*F*_c_^2^)/3
3087 reflections	(Δ/σ)_max_ < 0.001
264 parameters	Δρ_max_ = 0.12 e Å^−^^3^
0 restraints	Δρ_min_ = −0.18 e Å^−^^3^

### Special details

**Table:**

Geometry. All e.s.d.'s (except the e.s.d. in the dihedral angle between two l.s. planes) are estimated using the full covariance matrix. The cell e.s.d.'s are taken into account individually in the estimation of e.s.d.'s in distances, angles and torsion angles; correlations between e.s.d.'s in cell parameters are only used when they are defined by crystal symmetry. An approximate (isotropic) treatment of cell e.s.d.'s is used for estimating e.s.d.'s involving l.s. planes.
Refinement. Refinement of *F*^2^ against ALL reflections. The weighted *R*-factor *wR* and goodness of fit *S* are based on *F*^2^, conventional *R*-factors *R* are based on *F*, with *F* set to zero for negative *F*^2^. The threshold expression of *F*^2^ > σ(*F*^2^) is used only for calculating *R*-factors(gt) *etc*. and is not relevant to the choice of reflections for refinement. *R*-factors based on *F*^2^ are statistically about twice as large as those based on *F*, and *R*- factors based on ALL data will be even larger.

### Fractional atomic coordinates and isotropic or equivalent isotropic displacement parameters (Å^2^)

**Table:**

	*x*	*y*	*z*	*U*_iso_*/*U*_eq_	
C1	0.4797 (2)	0.37371 (19)	0.41816 (10)	0.0532 (5)	
H1	0.5236	0.4447	0.4370	0.064*	
C2	0.3360 (2)	0.3804 (2)	0.39415 (11)	0.0661 (6)	
H2	0.2837	0.4552	0.3973	0.079*	
C3	0.2704 (2)	0.2767 (3)	0.36573 (12)	0.0748 (7)	
H3	0.1739	0.2812	0.3493	0.090*	
C4	0.3477 (2)	0.1667 (3)	0.36166 (12)	0.0776 (8)	
H4	0.3033	0.0962	0.3426	0.093*	
C5	0.4916 (2)	0.1600 (2)	0.38590 (10)	0.0580 (6)	
H5	0.5433	0.0850	0.3827	0.070*	
C6	0.55940 (18)	0.26319 (17)	0.41470 (8)	0.0405 (5)	
C7	0.71658 (18)	0.25468 (16)	0.44225 (8)	0.0374 (4)	
H7	0.7559	0.1752	0.4268	0.045*	
C8	0.72666 (19)	0.24617 (17)	0.51335 (8)	0.0394 (4)	
C9	0.6212 (2)	0.26862 (18)	0.55446 (9)	0.0493 (5)	
H9	0.5270	0.2967	0.5429	0.059*	
C10	0.85246 (19)	0.20426 (17)	0.55141 (8)	0.0402 (4)	
C11	0.8153 (2)	0.20311 (18)	0.61463 (9)	0.0489 (5)	
C12	0.9142 (3)	0.1637 (2)	0.66314 (10)	0.0639 (6)	
H12	0.8885	0.1626	0.7049	0.077*	
C13	1.0503 (3)	0.1267 (2)	0.64757 (11)	0.0681 (6)	
H13	1.1183	0.1007	0.6795	0.082*	
C14	1.0901 (2)	0.1267 (2)	0.58488 (11)	0.0566 (6)	
C15	0.9929 (2)	0.16574 (17)	0.53660 (9)	0.0460 (5)	
H15	1.0197	0.1666	0.4950	0.055*	
C16	1.2814 (2)	0.0968 (2)	0.51545 (13)	0.0767 (7)	
H16A	1.2214	0.0460	0.4866	0.115*	
H16B	1.3809	0.0682	0.5160	0.115*	
H16C	1.2763	0.1834	0.5020	0.115*	
C17	0.80985 (17)	0.35843 (17)	0.41750 (8)	0.0378 (4)	
C18	0.8511 (2)	0.46889 (19)	0.44561 (9)	0.0502 (5)	
H18	0.8282	0.4933	0.4860	0.060*	
C19	0.94249 (19)	0.47413 (18)	0.35062 (9)	0.0445 (5)	
C20	0.86769 (17)	0.35972 (16)	0.35638 (8)	0.0365 (4)	
C21	0.86324 (18)	0.27284 (18)	0.30657 (8)	0.0422 (5)	
H21	0.8142	0.1964	0.3095	0.051*	
C22	0.9332 (2)	0.30362 (19)	0.25334 (9)	0.0468 (5)	
C23	1.0038 (2)	0.4195 (2)	0.24795 (10)	0.0566 (6)	
H23	1.0473	0.4387	0.2106	0.068*	
C24	1.0106 (2)	0.5050 (2)	0.29604 (10)	0.0571 (6)	
H24	1.0592	0.5815	0.2924	0.069*	
C25	0.8751 (4)	0.1091 (3)	0.20263 (14)	0.1214 (14)	
H25A	0.9235	0.0574	0.2348	0.182*	
H25B	0.8804	0.0692	0.1621	0.182*	
H25C	0.7744	0.1199	0.2116	0.182*	
N1	0.6733 (2)	0.24406 (16)	0.61507 (8)	0.0585 (5)	
H1A	0.6248	0.2529	0.6483	0.070*	
N2	0.93115 (18)	0.53917 (16)	0.40619 (8)	0.0568 (5)	
H2A	0.9683	0.6122	0.4148	0.068*	
O1	1.23040 (17)	0.08657 (16)	0.57707 (8)	0.0778 (5)	
O2	0.94253 (17)	0.22539 (15)	0.20173 (7)	0.0693 (5)	

### Atomic displacement parameters (Å^2^)

**Table:**

	*U*^11^	*U*^22^	*U*^33^	*U*^12^	*U*^13^	*U*^23^
C1	0.0506 (12)	0.0478 (13)	0.0616 (14)	0.0015 (10)	0.0059 (10)	−0.0035 (10)
C2	0.0535 (13)	0.0641 (16)	0.0804 (17)	0.0134 (11)	0.0026 (11)	−0.0016 (13)
C3	0.0444 (12)	0.092 (2)	0.0867 (18)	0.0084 (13)	−0.0066 (11)	−0.0137 (15)
C4	0.0510 (13)	0.0825 (19)	0.097 (2)	−0.0035 (13)	−0.0095 (12)	−0.0342 (15)
C5	0.0470 (11)	0.0568 (14)	0.0701 (15)	0.0024 (10)	0.0022 (10)	−0.0191 (11)
C6	0.0387 (10)	0.0447 (12)	0.0390 (11)	−0.0023 (8)	0.0090 (8)	−0.0032 (8)
C7	0.0385 (9)	0.0368 (10)	0.0376 (11)	−0.0013 (8)	0.0088 (8)	−0.0050 (8)
C8	0.0442 (10)	0.0363 (11)	0.0390 (11)	−0.0053 (8)	0.0115 (8)	−0.0025 (8)
C9	0.0522 (11)	0.0499 (12)	0.0472 (13)	−0.0019 (9)	0.0134 (10)	−0.0004 (9)
C10	0.0504 (11)	0.0320 (10)	0.0387 (11)	−0.0068 (8)	0.0072 (8)	−0.0012 (8)
C11	0.0643 (13)	0.0415 (12)	0.0419 (12)	−0.0069 (10)	0.0118 (10)	0.0000 (9)
C12	0.0972 (18)	0.0559 (14)	0.0384 (12)	−0.0070 (13)	0.0021 (12)	0.0074 (10)
C13	0.0833 (17)	0.0560 (15)	0.0626 (16)	0.0029 (12)	−0.0130 (13)	0.0122 (12)
C14	0.0611 (13)	0.0438 (13)	0.0641 (15)	−0.0001 (10)	−0.0033 (11)	0.0032 (10)
C15	0.0514 (11)	0.0403 (11)	0.0464 (12)	−0.0042 (9)	0.0046 (9)	−0.0017 (9)
C16	0.0547 (13)	0.0661 (17)	0.111 (2)	0.0008 (11)	0.0160 (14)	0.0084 (15)
C17	0.0364 (9)	0.0403 (11)	0.0372 (11)	−0.0024 (8)	0.0056 (8)	−0.0028 (8)
C18	0.0574 (12)	0.0516 (13)	0.0427 (11)	−0.0084 (10)	0.0122 (9)	−0.0075 (10)
C19	0.0474 (10)	0.0405 (11)	0.0463 (12)	−0.0032 (9)	0.0079 (9)	0.0011 (9)
C20	0.0341 (9)	0.0378 (11)	0.0379 (11)	−0.0009 (8)	0.0047 (7)	0.0008 (8)
C21	0.0402 (10)	0.0454 (12)	0.0418 (11)	−0.0063 (8)	0.0089 (8)	−0.0014 (9)
C22	0.0490 (11)	0.0546 (13)	0.0379 (11)	−0.0024 (9)	0.0112 (9)	−0.0024 (10)
C23	0.0662 (13)	0.0579 (14)	0.0483 (13)	−0.0051 (11)	0.0236 (10)	0.0102 (11)
C24	0.0662 (13)	0.0416 (13)	0.0659 (15)	−0.0092 (10)	0.0223 (11)	0.0068 (11)
C25	0.167 (3)	0.118 (3)	0.086 (2)	−0.081 (2)	0.067 (2)	−0.0612 (19)
N1	0.0740 (12)	0.0639 (12)	0.0403 (11)	−0.0035 (9)	0.0243 (9)	−0.0015 (8)
N2	0.0704 (11)	0.0422 (10)	0.0591 (11)	−0.0166 (8)	0.0140 (9)	−0.0078 (9)
O1	0.0604 (10)	0.0750 (12)	0.0961 (14)	0.0174 (8)	−0.0089 (9)	0.0057 (9)
O2	0.0840 (11)	0.0779 (11)	0.0491 (9)	−0.0194 (9)	0.0296 (8)	−0.0157 (8)

### Geometric parameters (Å, °)

**Table:**

C1—C2	1.382 (3)	C14—C15	1.374 (3)
C1—C6	1.384 (3)	C15—H15	0.9300
C1—H1	0.9300	C16—O1	1.417 (3)
C2—C3	1.372 (3)	C16—H16A	0.9600
C2—H2	0.9300	C16—H16B	0.9600
C3—C4	1.369 (3)	C16—H16C	0.9600
C3—H3	0.9300	C17—C18	1.356 (3)
C4—C5	1.385 (3)	C17—C20	1.429 (2)
C4—H4	0.9300	C18—N2	1.366 (2)
C5—C6	1.379 (3)	C18—H18	0.9300
C5—H5	0.9300	C19—N2	1.373 (2)
C6—C7	1.520 (2)	C19—C24	1.386 (3)
C7—C8	1.505 (2)	C19—C20	1.401 (2)
C7—C17	1.506 (2)	C20—C21	1.398 (2)
C7—H7	0.9800	C21—C22	1.371 (3)
C8—C9	1.362 (3)	C21—H21	0.9300
C8—C10	1.433 (3)	C22—O2	1.378 (2)
C9—N1	1.365 (3)	C22—C23	1.395 (3)
C9—H9	0.9300	C23—C24	1.361 (3)
C10—C11	1.402 (3)	C23—H23	0.9300
C10—C15	1.404 (3)	C24—H24	0.9300
C11—N1	1.371 (3)	C25—O2	1.379 (3)
C11—C12	1.389 (3)	C25—H25A	0.9600
C12—C13	1.367 (3)	C25—H25B	0.9600
C12—H12	0.9300	C25—H25C	0.9600
C13—C14	1.398 (3)	N1—H1A	0.8600
C13—H13	0.9300	N2—H2A	0.8600
C14—O1	1.373 (2)		
			
C2—C1—C6	121.1 (2)	C14—C15—H15	120.7
C2—C1—H1	119.4	C10—C15—H15	120.7
C6—C1—H1	119.4	O1—C16—H16A	109.5
C3—C2—C1	120.0 (2)	O1—C16—H16B	109.5
C3—C2—H2	120.0	H16A—C16—H16B	109.5
C1—C2—H2	120.0	O1—C16—H16C	109.5
C4—C3—C2	119.8 (2)	H16A—C16—H16C	109.5
C4—C3—H3	120.1	H16B—C16—H16C	109.5
C2—C3—H3	120.1	C18—C17—C20	106.26 (15)
C3—C4—C5	120.2 (2)	C18—C17—C7	128.73 (16)
C3—C4—H4	119.9	C20—C17—C7	124.96 (15)
C5—C4—H4	119.9	C17—C18—N2	110.36 (17)
C6—C5—C4	120.9 (2)	C17—C18—H18	124.8
C6—C5—H5	119.5	N2—C18—H18	124.8
C4—C5—H5	119.5	N2—C19—C24	131.17 (18)
C5—C6—C1	118.03 (17)	N2—C19—C20	107.18 (15)
C5—C6—C7	120.69 (17)	C24—C19—C20	121.65 (18)
C1—C6—C7	121.27 (17)	C21—C20—C19	119.64 (16)
C8—C7—C17	113.07 (14)	C21—C20—C17	132.98 (16)
C8—C7—C6	112.52 (14)	C19—C20—C17	107.37 (15)
C17—C7—C6	111.53 (15)	C22—C21—C20	118.07 (17)
C8—C7—H7	106.4	C22—C21—H21	121.0
C17—C7—H7	106.4	C20—C21—H21	121.0
C6—C7—H7	106.4	C21—C22—O2	124.35 (18)
C9—C8—C10	105.80 (17)	C21—C22—C23	121.32 (18)
C9—C8—C7	128.92 (17)	O2—C22—C23	114.32 (17)
C10—C8—C7	125.19 (15)	C24—C23—C22	121.57 (18)
C8—C9—N1	110.34 (18)	C24—C23—H23	119.2
C8—C9—H9	124.8	C22—C23—H23	119.2
N1—C9—H9	124.8	C23—C24—C19	117.70 (19)
C11—C10—C15	119.51 (18)	C23—C24—H24	121.2
C11—C10—C8	107.68 (16)	C19—C24—H24	121.2
C15—C10—C8	132.80 (17)	O2—C25—H25A	109.5
N1—C11—C12	131.56 (19)	O2—C25—H25B	109.5
N1—C11—C10	106.98 (17)	H25A—C25—H25B	109.5
C12—C11—C10	121.46 (19)	O2—C25—H25C	109.5
C13—C12—C11	118.0 (2)	H25A—C25—H25C	109.5
C13—C12—H12	121.0	H25B—C25—H25C	109.5
C11—C12—H12	121.0	C9—N1—C11	109.19 (16)
C12—C13—C14	121.7 (2)	C9—N1—H1A	125.4
C12—C13—H13	119.2	C11—N1—H1A	125.4
C14—C13—H13	119.2	C18—N2—C19	108.83 (16)
O1—C14—C15	124.7 (2)	C18—N2—H2A	125.6
O1—C14—C13	114.60 (19)	C19—N2—H2A	125.6
C15—C14—C13	120.7 (2)	C14—O1—C16	116.91 (17)
C14—C15—C10	118.66 (19)	C22—O2—C25	118.42 (16)
			
C6—C1—C2—C3	−0.7 (3)	C8—C10—C15—C14	−178.15 (19)
C1—C2—C3—C4	0.4 (4)	C8—C7—C17—C18	−28.6 (3)
C2—C3—C4—C5	−0.2 (4)	C6—C7—C17—C18	99.3 (2)
C3—C4—C5—C6	0.3 (4)	C8—C7—C17—C20	154.25 (16)
C4—C5—C6—C1	−0.5 (3)	C6—C7—C17—C20	−77.8 (2)
C4—C5—C6—C7	178.92 (19)	C20—C17—C18—N2	−0.1 (2)
C2—C1—C6—C5	0.7 (3)	C7—C17—C18—N2	−177.62 (17)
C2—C1—C6—C7	−178.74 (18)	N2—C19—C20—C21	−179.02 (16)
C5—C6—C7—C8	−104.3 (2)	C24—C19—C20—C21	1.2 (3)
C1—C6—C7—C8	75.1 (2)	N2—C19—C20—C17	0.3 (2)
C5—C6—C7—C17	127.43 (19)	C24—C19—C20—C17	−179.42 (17)
C1—C6—C7—C17	−53.1 (2)	C18—C17—C20—C21	179.09 (19)
C17—C7—C8—C9	113.9 (2)	C7—C17—C20—C21	−3.3 (3)
C6—C7—C8—C9	−13.6 (3)	C18—C17—C20—C19	−0.2 (2)
C17—C7—C8—C10	−70.0 (2)	C7—C17—C20—C19	177.49 (16)
C6—C7—C8—C10	162.53 (17)	C19—C20—C21—C22	0.0 (3)
C10—C8—C9—N1	0.3 (2)	C17—C20—C21—C22	−179.20 (18)
C7—C8—C9—N1	176.99 (16)	C20—C21—C22—O2	177.46 (17)
C9—C8—C10—C11	0.2 (2)	C20—C21—C22—C23	−1.7 (3)
C7—C8—C10—C11	−176.63 (16)	C21—C22—C23—C24	2.3 (3)
C9—C8—C10—C15	179.1 (2)	O2—C22—C23—C24	−176.93 (19)
C7—C8—C10—C15	2.2 (3)	C22—C23—C24—C19	−1.1 (3)
C15—C10—C11—N1	−179.67 (17)	N2—C19—C24—C23	179.6 (2)
C8—C10—C11—N1	−0.7 (2)	C20—C19—C24—C23	−0.7 (3)
C15—C10—C11—C12	−0.5 (3)	C8—C9—N1—C11	−0.7 (2)
C8—C10—C11—C12	178.51 (18)	C12—C11—N1—C9	−178.2 (2)
N1—C11—C12—C13	179.5 (2)	C10—C11—N1—C9	0.9 (2)
C10—C11—C12—C13	0.5 (3)	C17—C18—N2—C19	0.3 (2)
C11—C12—C13—C14	−0.6 (3)	C24—C19—N2—C18	179.3 (2)
C12—C13—C14—O1	−179.8 (2)	C20—C19—N2—C18	−0.4 (2)
C12—C13—C14—C15	0.7 (3)	C15—C14—O1—C16	5.7 (3)
O1—C14—C15—C10	179.89 (18)	C13—C14—O1—C16	−173.79 (19)
C13—C14—C15—C10	−0.7 (3)	C21—C22—O2—C25	1.3 (3)
C11—C10—C15—C14	0.6 (3)	C23—C22—O2—C25	−179.6 (2)

### Hydrogen-bond geometry (Å, °)

**Table:**

*Cg*2, *Cg*3, *Cg*4 and *Cg*5 are the centroids of the N2/C17–C20, C1–C6, C10–C15 and C19–C24 rings, respectively

**Table:**

*D*—H···*A*	*D*—H	H···*A*	*D*···*A*	*D*—H···*A*
N1—H1A···O2^i^	0.86	2.09	2.904 (2)	158
C25—H25B···Cg2^ii^	0.96	2.95	3.515 (3)	119
C16—H16C···Cg3^iii^	0.96	2.90	3.508 (3)	122
N2—H2A···Cg4^iv^	0.86	2.49	3.3149 (19)	160
C3—H3···Cg5^v^	0.93	2.60	3.470 (2)	157

Symmetry codes: (i) *x*−1/2, −*y*+1/2, *z*+1/2; (ii) −*x*+3/2, *y*−1/2, −*z*+1/2; (iii) *x*+1, *y*, *z*; (iv) −*x*+2, −*y*+1, −*z*+1; (v) *x*−1, *y*, *z*.
